# Etiology, pathogenesis, and management of angiosarcoma associated with implants and foreign body: Clinical cases and research updates

**DOI:** 10.1097/MD.0000000000037932

**Published:** 2024-05-03

**Authors:** Ramy Samargandi

**Affiliations:** a Department of Orthopedic Surgery, Faculty of Medicine, University of Jeddah, Jeddah, Saudi Arabia; b Service de Chirurgie Orthopédique et Traumatologique, CHRU Trousseau, Faculté de Médecine de Tours, Université de Tours, Chambray-les-Tours, France.

**Keywords:** angiosarcoma, biomedical implants, foreign body tumorigenesis, metastasis, orthopedic prosthesis, vascular prosthesis

## Abstract

Angiosarcomas are rare and highly malignant soft tissue sarcomas originating from endothelial cells lining the lymphatic or vascular system. While they predominantly emerge from (sub)cutaneous regions, occurrences have been reported throughout the body. The etiology of angiosarcoma remains elusive in most clinical cases. Nevertheless, several prognosis risk factors play a pivotal role, including chronic lymphedema, therapeutic irradiation, environmental carcinogens, familial syndromes, and the presence of foreign materials like metallic objects and biomedical implants. Despite evidence implicating retained foreign material in angiosarcoma development, understanding its prognosis and pathogenesis remains limited. The pathogenesis of angiosarcoma appears to involve a complex interplay of chronic inflammation, tissue remodeling, and genetic factors that create a conducive microenvironment for malignant transformation. Management of these sarcomas remains challenging due to their infiltrative nature owing to the high chance of metastasis and local recurrence. The primary treatment modalities currently include surgery, radiotherapy, and chemotherapy, but recent advances in targeted immunotherapy and gene therapy hold promise for more effective approaches. This comprehensive review delves into the potential etiological and pathogenic roles of foreign materials, such as metallic objects, biomedical implants, and biomaterials, in the development of angiosarcoma. Further research into the underlying molecular mechanisms could provide valuable insights for tailored management and developing novel targeted therapeutic strategies.

## 1. Introduction

Sarcomas are a rare type of tumor that originates from malignant transformation of cells that make up the connective tissue (bone and supporting soft tissue). They are majorly categorized as bone sarcoma and soft tissue sarcoma. Till now, more than 50 different types of soft tissue sarcoma have been characterized.^[1]^ Angiosarcoma (AS) is a rare subtype of soft tissue sarcoma, constituting up to 1% to 2% of sarcoma cases diagnosed in humans. The term angiosarcoma is derived from 2 Greek words, “angio” (vessel) and “sarcoma” (flesh); thus, AS is described as the malignant transformation of endothelial cells lining the vascular or lymphatic channels.^[2,3]^ AS develops primarily (~60–70% cases) at the cutaneous (skin) and subcutaneous (superficial soft tissue) regions of the body. These types of ASs are referred to as “cutaneous angiosarcoma” and are predominantly observed in the scalp regions of the head and neck. Other than this, the development of AS has also been reported in deep soft tissues of the breast and visceral organs, including the lung, spleen, liver, bone, and retroperitoneum.^[4–6]^ AS are commonly diagnosed in adults and elderly patients with an incidence rate of ~1.5 per million people per year. ASs are aggressive and infiltrative, with high chances of local recurrence and greater metastatic potential. The metastasis rate of ~16% to 44% has been observed in patients with a poor median survivability rate ranging from ~1 to 3 years. Epidemiologically, AS does not show any biases for either of the 2 sexes. However, the cutaneous type occurring at the neck and head regions is detected more commonly in elderly men.^[3,5,7]^

The etiology of the disease is not well defined, and the diagnosis remains challenging due to rarity and poor prognosis. AS may arise spontaneously *de novo* without predisposing conditions or systemic syndromes. Nevertheless, several reports have identified potential predisposing risk factors such as radiation therapy, chronic lymphedema, familial genetic syndromes, environmental carcinogens, and retained foreign body material responsible for the pathogenesis of AS.^[1,2,8]^ Various reports have also highlighted the association of AS with the presence of foreign body material in human and animal models.^[9–11]^ These cases were related to the development of AS at sites where vascular grafts, and orthopedic implants, such as prosthesis and metallic plates were present.^[12–14]^ Other reports also described the presence of metallic objects (shell splinter and bullet), surgical sponge/gauze, bone wax, cardiac defibrillator, and biomaterials at sites presenting AS proliferation.^[15–17]^ Chronic inflammation around these foreign materials could probably potentiate the pathogenic progression to the malignant transformation of surrounding soft tissues. However, a direct connection between the development of AS and the presence of foreign material at those sites has not been established in humans. This is the first study where we review the cases related to the development of AS in association with retained foreign body material and biomedical implants, unveiling their potential involvement in the pathogenesis and etiological correlation to clinical and histopathological findings.

## 2. Etiology, diagnostic imaging, histopathology and pathogenesis of angiosarcoma

The rarity, aggressiveness, and highly malignant nature of AS with poor survival rate posttreatment has drawn serious scientific attention with fundamental advances in understanding the disease pathology in the past decade. The exact etiology of AS remains multifaceted and, in many cases, elusive. While several potential risk factors have been identified, a direct pathogenic mechanism is often challenging to pinpoint.^[1,2]^ Radiation exposure emerges as a prominent risk factor in most cases diagnosed with AS. In the past 30 years, the incidence of AS has significantly increased, possibly due to many patients undergoing radiation therapy, advancement in differential diagnosis, or widespread medical awareness of the disease.^[18]^ Prolonged exposure to environmental carcinogens (arsenic, vinyl chloride, and thorium dioxide), particularly in occupational settings, has also been implicated in AS developments.^[19]^ Other risk factors tightly linked to AS are predisposing familial genetic syndromes (Klippel-Trenaunay syndrome, Maffucci syndrome, and neurofibromatosis), genetic mutations in those involving TP53 gene and BRCA1 or BRCA2, and chronic lymphedema (Steward-Treves syndrome).^[20–23]^ Although most angiosarcomas arise sporadically, the interplay of genetic, environmental, and iatrogenic factors is increasingly recognized as influential in its etiology.

Foreign body tumorigenesis is a well-established phenomenon that has been experimentally validated in laboratory animals.^[9–11,24]^ However, due to poor prognosis, direct evidence of such an event in humans remains enigmatic. The tested potentials of foreign body in evoking sarcoma development has enlisted them as an independent etiological risk factor. Several reports in the literature have identified AS development vicinal to a retained foreign material in the body with no history of exposure to radiation, carcinogens, or genetic syndromes. The vicinal association of the foreign body to the AS development indicates their significant involvement in the pathogenesis of the disease. Previous reports have described various foreign materials that could be strongly linked to AS. These retained foreign materials could belong to either of the categories, such as metallic objects (Shell splinter, bullet, surgical sponge/gauze, or non-arthroplasty implants), orthopedic and vascular prosthetic implants.^[12–15,17]^

Diagnostic differentiation of angiosarcoma from other malignant carcinomas presents a challenging situation due to nonspecific clinical presentations and disease rarity. The multifocal ubiquitous clinical presentation with symptoms such as vomiting, abdominal discomfort, nausea, and bowel distension adds to the difficulty in early diagnostics. AS primarily occurs at the face, scalp, breast, and other deep soft tissues. AS can initially present as a benign lesion or chronic inflammation, but tissue filtration, edema, ulceration, hemorrhage, and fibrosis may propagate with increasing tumor size. The tumorous palpable lesion may grow up to 20 cm or more in size. The tissue infiltration propagates as deep tissue and visceral lesions transform into tumor mass, resulting in extreme pain, jaundice, fatigue, and discomfort. With increasing aggressive and malignancy, AS can metastasize haematogenously, infiltrating the lungs, and may manifest as pleural effusion or dyspnea. Advanced metastasis may spread multifocally, infecting the liver, lymph nodes, bone, brain, spleen, and other soft tissues.^[2,3,25–27]^ In 15% of the cases, the patient is diagnosed with the disease at the metastatic state, and in ~70% to 80% of cases, local recurrences have been described posttreatment. Diagnostic imaging techniques may aid in initial preoperative diagnosis and provide definitive clinical suspicions for further course of treatment.^[28]^ Ultrasound, CT (positron emission tomography-computed tomography [PET-CT]), and MRI (magnetic resonance imaging) are the most commonly used diagnostic imaging procedures. These imaging techniques may provide the necessary clinical assessment of treatment response, staging the metastasis of the disease and detecting the calcifications to identify bone involvement.^[27,29,30]^

**Figure 1.**
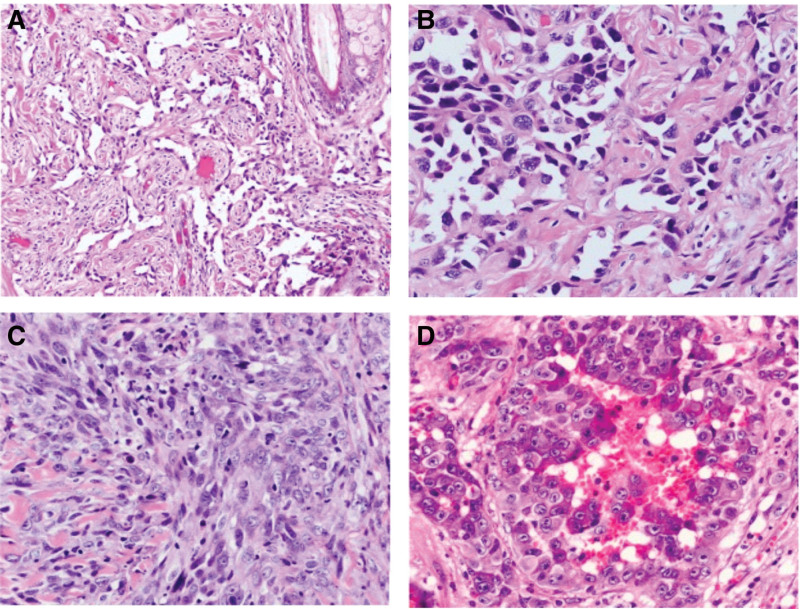
Histopathological representation of angiosarcoma. Photomicrograph of hematoxylin and eosin (H&E) stained histological samples demonstrating; low-grade angiosarcoma with irregular and anastomosing vascular channels lined by atypical endothelial cells, (A) original magnification ×200 and (B) original magnification ×400. (C) Poorly differentiated high-grade angiosarcoma marked by continuous proliferating sheets of spindle tumor cells lacking clear vasoformation, original magnification ×400. (D) High-grade angiosarcoma showing distinct epithelioid appearance with an abundance of amphophilic-lightly eosinophilic cytoplasm, original magnification ×400. This figure has been reproduced with permission from Wang et al *Oncol Lett* (2017) 14:5370–8.^[7]^

Diagnostic imaging features are relatively nonspecific, presenting challenges in the differential diagnosis of AS from other tumors. Thus, histological and immuno-histochemical identification of the tumor specimen is paramount for differential diagnosis. Histopathological samples for diagnostic analysis could be obtained by percutaneous needle biopsy, open biopsy, frozen biopsy or from tumor resection. AS can present histologically diverse features with a spectrum ranging from highly differentiated malignant to poorly differentiated benign forms. Differentiation of the benign and malignant forms under light microscopy could be challenging. Due to their infiltration nature, the distinguishing border between the proliferative AS and normal tissue remains perplexing. AS is hallmarked by the presence of abnormal, pleomorphic, and malignant endothelial cells that may appear rounded, fusiform, epithelioid, or polygonal. The poorly differentiated AS may appear as sheets of spindle-shaped, polygonal, or epithelioid morphology with poorly formed vascular spaces and higher mitotic activity and marked by areas of hemorrhage and necrosis (Fig. 1A and B). The well-differentiated malignant forms present as numerous abnormal endothelial cells with definitive vascular sinusoids that dissect through the collagen bundles and often are associated with monocyte infiltration (Fig. 1C and D). The highly malignant and aggressive form may present a more chaotic architecture organized as multilayered abnormal endothelial cells with poorly defined vascular spaces, atypical nuclei, and papillary-like projections.^[7,30–32]^

**Figure 2.**
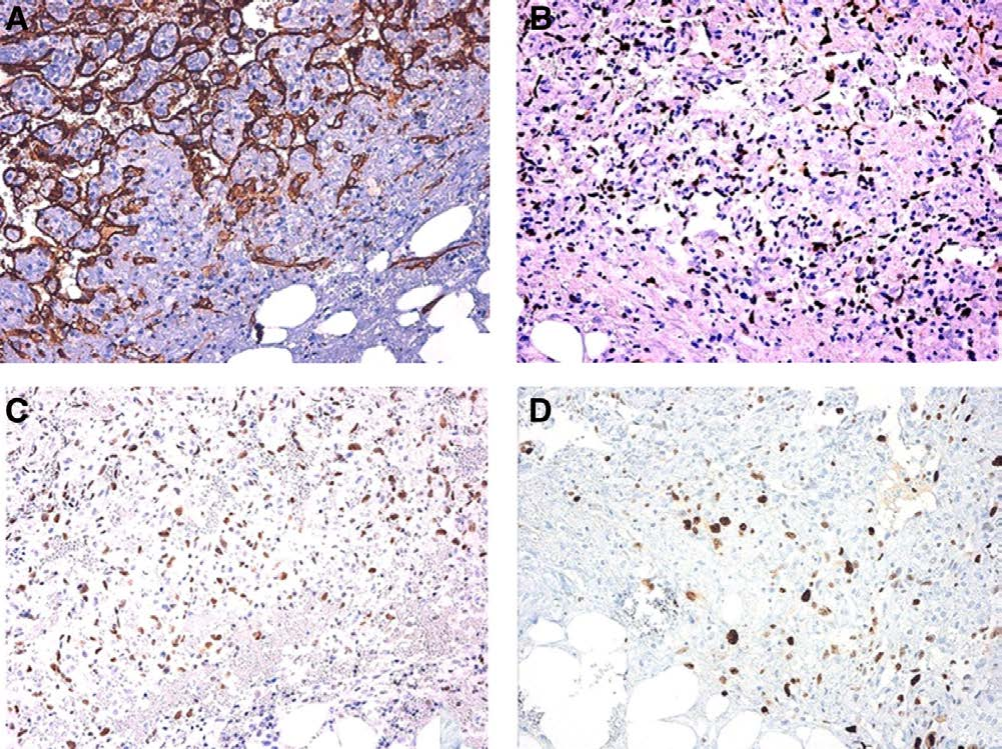
Immunohistopathological representation of angiosarcoma. Photomicrograph of histological samples showing positivity for immunostains reactive against (A) CD31, (B) ERG, (C) FLI1, and (D) Ki67. Original magnification ×200. This figure has been reproduced with permission from Presacco et al *BMC Surg* (2020) 20:291.^[12]^

The poorly differentiated forms present diagnostic challenges due to heterogeneous cytological features and nonspecific characteristics similar to melanoma or anaplastic carcinoma. Thus, immunohistochemical analysis is necessary in the differential diagnosis of poorly differentiated AS types. AS typically expresses endothelial markers CD31, CD34, Von Willebrand factor, Factor-VIII related antigen, and vascular endothelial growth factor (VEGF). Cytogenetic positivity for the upregulation of FLI-1 and ERG transcription factors are also definitive markers for AS. CD31 and ERG expression are defined as gold standard markers for immunohistochemical identification of AS in the 2020 WHO classification (Fig. 2). Positivity for expression of CD31 and cytokeratin and negativity for CD34 markers are definitive for epithelioid AS, distinguishing them from poorly differentiated carcinomas. In cases of progressive tumor dedifferentiation, AS is distinguished from melanoma by negativity for melanocytic markers.^[7,31,32]^

Pathogenic progression of AS remains enigmatic, and the mechanism is poorly understood due to poor prognosis. Earlier animal studies have well-established the tumorigenic potentials of foreign bodies.^[9–11,24,33]^ It has been hypothesized that the solid-state carcinogenesis associated with the inert foreign material is mediated by fibrous capsule formation. Introducing the foreign material into the body may induce local tissue injury and blood extravasation that triggers the chronic inflammatory response pathway. This leads to granulation tissue response in which the site of the foreign body is infiltrated with neutrophils, monocytes, macrophages, and matrix proteins, adhering them to the inflammatory site. AS initially can appear as inflammatory lesions and, if ignored, may eventually progress into a tumor mass with metastatic potentials. The long-standing granulation tissue response, venous ulceration, and hemorrhage further prognoses to the fibrotic stage through extensive tissue fibrosis evolving into a mature fibrous capsule surrounding the implant. The abnormal malignant mesenchymal cells undergo remodeling into neoplastic endothelial cells and fibroblast sequestered with vascular elements.^[24,34]^ However, direct evidence linking the foreign body tumorigenic potential to AS development remains baffled and, therefore, necessitates a detailed exploration of the molecular pathogenesis pathway.

## 3. Foreign body tumorigenesis: metallic and other biomedical objects linked to angiosarcoma

Foreign material accidentally appearing in the body or incorporated for medical reasons may elicit a chronic inflammatory response or fibrogenesis, eventually culminating in sarcomatous tissue development. These sarcomatous tissues, if not diagnosed early, may further transform into a malignant state and advance to the metastatic phase, spreading across the body and affecting other organs. The highly infiltrative and metastatic characteristics of these sarcoma lead to higher morbidity and mortality rates in affected patients. Several cases of angiosarcoma associated with foreign bodies in humans have been reported and are available in the medical literature from the late 19th century. An etiological relationship linking AS to foreign bodies remains questionable, and the pathogenic process involved tends to be underexplored. However, various experimental investigations on laboratory animals have explored the potential of foreign body materials in eliciting the development of sarcomas.^[9–11,24]^ The evidence from these exploratory animal experiments has identified the tumorigenic capabilities of the foreign body in the vicinal tissues and has opened new research avenues for detailed investigation.

In this section, we review all the case reports and case series where histological evidence has described the proliferation of angiosarcoma in the vicinity of retained foreign material fortuitously or incorporated surgically to treat clinical conditions. Foreign bodies observed in humans could result from various scenarios. Metallic objects are one of the categories of foreign bodies observed in humans. They may gain access to human tissues through wound injury due to accidents, surgical negligence/accidental loss, or otherwise incorporated as a part of orthopedic surgical procedures in certain clinical conditions. Over time, these metallic objects may induce chronic inflammation in surrounding tissues and trigger infiltration of inflammatory molecules and immune reactive cells, resulting in edema and tissue lesions. Later on, these lesions may set the stage for proliferation and sarcomatous transformation, forming a palpable tumor mass in the body. A detailed list of all the clinical cases is summarized in Table 1, reported from the late 19th century till now, where a potential involvement of metallic foreign body is suspected in the development of AS.

**Table 1:** Review of literature for the cases of metallic and other foreign materials associated with AS development.

Author	Year	Age/sex	Foreign material	Interval to AS development (yr)	Tumor site	Tumor status	Treatment/management	Outcome
McDougall^[35]^	1956	42/M	Metal plate and screws	30	Upper arm	Metastasized to lungs	Surgery, radiation therapy	No local recurrence and died 14 mo after diagnosis
Dube et al^[36]^	1972	84/M	Metal plate and screws	27	Left tibia	Metastasized to lung, diaphragm, and brain	Surgery	Local recurrences and died within 1 yr of treatment
Hayman et al^[37]^	1983	86/M	Metal grenade fragment	63	Right axilla	No metastasis reported	Surgery, chemotherapy	Local recurrences and died 2 yr after diagnosis
Jennings et al^[38]^	1988	79/M	Bullet	54	Right thigh	Metastasized to lungs	Surgery	Local recurrences and died within 3 mo of treatment
Jennings et al^[38]^	1988	70/F	Sponge	20	Peritoneum	Metastasized to lungs and liver	Surgery	Local recurrences and died within 3 mo of treatment
Jennings et al^[38]^	1988	64/F	Bone wax	30	Right tibia	Metastasized to lung and skin	Surgery, chemotherapy	Local recurrences and died within 1 yr of treatment
Ben-Izhak et al^[39]^	1991	70/F	Sponge	25	Rectum	Metastasized to ileum and bone	Surgery, radiation therapy	Local recurrences and died within 2 mo of treatment
Schneider et al^[40]^	1997	ND/M	Bullet	46	Left tibia	Metastasized to lung	Amputation of leg	Local recurrences and died within 2 mo of treatment
Cokelaere et al^[15]^	2001	67/M	Gauze sponge	38	Splenic capsule	No metastasis reported	Surgery	Died within 2 wk of first surgery
Ferrari et al^[41]^	2001	48/M	Kuntscher nail	31	Left distal thigh	No metastasis reported	Surgery	No recurrence, patient was well and alive 3 yr after surgery
Mcdonald et al^[42]^	2002	70/M	Metal plate and screws	52	Left thigh	Metastasized to iliac and para-aortic nodes	Surgery, postoperative chemotherapy	Local recurrences and died within 1 yr of treatment
Mcdonald et al^[42]^	2002	71/M	Metal plate and screws	41	Left thigh	No metastasis reported	Surgery	No recurrence, patient was well and alive for 10 yr posttreatment
Keymeulen et al^[43]^	2004	73/M	Gauze sponge	38	Splenic capsule	No metastasis reported	Surgery	Died within 1 mo of treatment
Joo et al^[44]^	2005	61/M	Gauze	20	Distal ileum	Metastasized to liver	Surgery	Multiple local recurrences and died within 2 mo of treatment
Teltzrow et al^[45]^	2006	84/M	Shrapnel/shell splinter	60	Face	General metastasis	Surgery	Local recurrences and died within 3 mo of treatment
Park et al^[46]^	2012	62/M	Stainless steel nail	16	Distal femur	No metastasis reported	Surgery, chemotherapy	No local recurrence, and the patient died 21 mo after surgery

AS = angiosarcoma, F = female, M = male, ND = not disclosed.

While several cases of soft tissue sarcomas linked to foreign body tumorigenesis have been observed at the site of these retained foreign materials. A literature search through databases using keywords of angiosarcoma and foreign body/metallic objects identified 16 such reported cases where malignant and metastatic proliferation of angiosarcoma have been observed developing in the vicinity of a retained metallic foreign body. The retained metallic and other objects could be shell splinters, bullets, shrapnel, surgical sponge and gauze, incorporated metallic plates, screws, and bone wax used in orthopedic surgery, etc. Most of these cases were observed in adult men, with a few in adult women. A gender-based association should not be considered as these sarcomas have likely developed due to foreign body presence. Also, no such association of AS with gender has been reported in past literature. In these cases, the latency period (time lapse between the entry/installment of the foreign body and the identification of AS) ranges from 16 to 63 years.

Accidental loss of surgical sponge in the human body during surgery is a commonly observed negligence in hospitals and thus contributes to one of the entry modes for foreign body material. It is one of the most embarrassing hospital-based negligence cases that could cause dramatic, sophisticated clinical situations. Such a medical situation is termed *gossypiboma* or *textiloma.*^[47,48]^ The first case was described by Wilson in 1884,^[49]^ and thereafter, an incidence rate of 1 in 300 to 1 in 3000 has been observed in past reports across all surgical procedures. *Gossypiboma* has been demonstrated to be associated with the development of severe clinical conditions involving various sarcomas or granuloma (pseudotumor).^[43,44,50]^ Five cases of *gossypiboma* associated with the proliferative development of AS have been observed in the literature search, pointing to the devastating outcome of such sheer medical negligence during surgery. All these *gossypiboma* cases reported linked to AS have been observed in patients who underwent abdominal surgery.

Metallic foreign material gains entry into the body accidentally as war shells or shell splinters or could be incorporated as non-arthroplasty implants during orthopedic surgery. McDougall, in 1956, reported the first case of high-grade vascular lesions suspected of haemangiosarcoma in the soft tissue samples excised from the humerus in the right arm, which was fixed with metallic plate and screws to heal a fracture incident 30 years ago.^[35]^ Later, similar cases of proliferative lytic vascular lesions identified as AS associated with metallic plate and screws fixation of the fracture were reported by Dube and Fisher^[36]^ and McDonald et al^[42]^ We could identify 4 cases from literature review where patients had retained shell splinter or bullet in various regions in the body during a war encounter in their life. These retained metallic fragments reacted with the surrounding tissue over time, resulting in lytic lesions and finally culminating in AS transformation.

Preoperative imaging techniques were used to detect the cause of pain and discomfort in patients, resulting in the identification of tender mass and lytic lesions and raising suspicion of sarcoma. Further categorization of the sarcomatous growth involved immunohistochemical analysis using samples from a core needle biopsy or excised tissue postoperatively. The tender mass identified in the above reported cases was resected through surgical procedures, and the patient was advised chemotherapy or radiotherapy based on the clinical conditions. However, in most of the cases, patients were readmitted with local recurrences of the tumor mass associated with pain and discomfort. Radiological imaging for detection of metastatic progression showed advanced metastasis to other body organs and tissues, resulting in aggravating severe conditions and death risk. The patient’s clinical status progressively deteriorated, resulting in death in most cases. Only 2 cases with non-arthroplasty implants were observed where the patient recovered posttreatment and was well and alive. In these cases, the decision for hemipelvectomy^[42]^ and hip articulation^[41]^ was taken instead of amputation before any signs of distant or local metastasis, which led to recovery posttreatment.

In all the cases listed in Table 1, no potential prognostic factors could be identified based on the patient’s medical history or any toxic exposure to carcinogens that could trigger AS development other than the proximal association of metallic foreign objects. Thus, the pathogenic association of metallic objects in the development of AS could plausibly assumed and therefore requires deep scientific exploration to understand disease prognosis.

## 4. Biomedical implant tumorigenesis: orthopedic prosthesis linked to angiosarcoma

Orthopedic prosthesis have been used extensively as implants for joint replacement after osteoarthritis or post-traumatic fractures. Various biomaterials have been used to construct orthopedic prostheses to provide nontoxic and inert characteristics. The first breakthrough implant material was Vitallium (Co, Cr, Mb) alloy, which was introduced in 1929 for producing orthopedic implants. Later on, it was replaced by titanium-based alloy material for long-term or lifelong usage due to its nontoxic and biologically inert properties. The load-bearing interface could be made up of metallic alloys or polyethylene and bone interface of titanium.^[33,51]^ The wear-off metallic particle from these implants has a well-documented ability to potentiate tumorigenesis in animal models, yet the mechanism remains enigmatic to researchers.^[52]^ However, considering the huge number of total hip arthroplasties, revisions, or partial hip arthroplasties, the occurrence of sarcomas proximal to these implants is rare but a well-recognized complication.

Although a direct etiological link between the development of sarcoma in humans and the presence of orthopedic implants has not been characterized. A considerable number of individual cases of orthopedic implant-associated sarcoma has been reported in the literature after identification of the first case in 1956.^[35]^ Though we could identify a few cases (N = 15) of AS associated with orthopedic implants after screening through the literature. In this study, we consider it worthwhile to outline the extent of the problem and delineate the possible clinicopathological features for better understanding and the necessity for a detailed investigation. We have enlisted all of the possible cases of arthroplasty implant-associated AS to date in Table 2. The latency period has been highly variable across each case, ranging from 1 to 31 years, with most cases having a latency period of >6 years. The cases reported by Weber^[53]^ and Lee et al^[14]^ had a lesser latency period because of the trauma (fall) after arthroplasty. Notably, a case of a 72-year-old male reported by Drexler et al^[56]^ in 2009 was diagnosed by AS of bone 15 months after total knee arthroplasty. In this case, the shorter latency period could probably result from the previous tibial plateau fracture managed by a fluted rod during the total knee arthroplasty. We do not observe a gender-based association of AS with orthopedic implants in all of the screened cases. Nearly equal cases were observed in both sexes, pointing to a direct implication of metallic implants in triggering malignant transformation.

**Table 2:** Review of literature for cases of orthopedic prosthesis associated with AS development.

Author	Year	Age/sex	Orthopedic implant	Interval to AS development (yr)	Tumor site	Tumor status	Treatment/management	Outcome
Weber^[53]^	1986	76/F	Total knee replacement	4.5	Left popliteal artery	Metastasized to lung, bone	Palliative chemotherapy	Died 6 wk after diagnosis
van der List et al^[54]^	1988	72/F	Total hip arthroplasty	11	Right hip	No metastasis reported	Surgery	ND
Mallick et al^[55]^	2009	84/F	Total hip arthroplasty	30	Right hip	No metastasis reported	Surgery, palliative radiotherapy	ND
Drexler et al^[56]^	2009	72/M	Total knee arthroplasty	1.25	Proximal tibia	No metastasis reported, lytic lesion observed in pelvis and proximal femur	Surgery, adjuvant radiotherapy	ND
Albert et al^[57]^	2009	85/F	Total knee arthroplasty	10	Right tibia	Metastasized to left hemithorax, lymph nodes	Surgery	Died 1 mo after mid-thigh amputation
Fabbri et al^[58]^	2010	80/M	Total hip arthroplasty	27	Right hip	Metastasized to lung	Surgery	Died shortly after surgery
Zhu et al^[59]^	2016	ND	Total hip arthroplasty	14	Left hip	No metastasis reported	Surgery	Died 9 wk after surgery
Agaimy et al^[60]^	2016	78/M	Total hip endoprosthesis	17	Left hip	No metastasis reported	No	Died shortly after diagnosis
Agaimy et al^[60]^	2016	55/M	Bilateral hip replacement	8	Left hemi-pelvis	Metastasized to lung	Surgery, radio-chemotherapy	Alive 17 mo after surgery
Terrando et al^[61]^	2018	75/M	Total hip arthroplasty	7	Right hip	Metastasized to lung	Hindquarter amputation, adjuvant chemotherapy	Alive with disease 7 mo after surgery
Terrando et al^[61]^	2018	74/M	Total hip arthroplasty	16	Left hip	Metastasized to lung	Hindquarter amputation, chemotherapy	Died 27 mo after surgery
Terrando et al^[61]^	2018	63/F	Total hip arthroplasty	31	Right hip	Metastasized to lung	Surgery, palliative chemotherapy	Died 5 mo after surgery
Terrando et al^[61]^	2018	80/M	Total hip arthroplasty	27	Right hip	Bilateral widespread metastasis	Hindquarter amputation	Died 4 mo after surgery
Terrando et al^[61]^	2018	76/F	Total hip arthroplasty	13	Right hip	Metastasized to lung	Surgery, chemotherapy	Died 9 mo after surgery
Lee et al^[14]^	2021	69/F	Total hip arthroplasty	1	Left hip	No metastasis reported	Radiotherapy	Died 40 d after diagnosis

AS = angiosarcoma, F = female, M = male, ND = not disclosed.

Another aspect that has been observed in these cases is that all of the patients are elderly and are more than 50 years old. Though the clinical circumstances for total hip arthroplasty were unavailable in most cases, osteoarthritis could be the primary reason, considering the elderly age. Except in cases reported by Weber^[53]^ and Zhu et al^[59]^ where bone infarct/enchondroma and giant cell tumor towards the left femoral head respectively being the reason for total hip arthroplasty (THA). Severe osteolysis around the prosthetic implants causing shortening of the skeletal component has been the major factor contributing to admission of patients with severe hip pain. In many cases, the complaint of the pain has been from the past few years. In most cases, severe osteolysis around the prosthetics had contributed to the aseptic cup loosening and a need for revision of the implant.

Summarily, the plausible pathogenic mechanism involved in the development of AS in arthroplasty implant patients is primarily linked to the severe periprosthetic osteolysis that gradually increased over time. The osteolysis resulted in the destabilization of the skeletal component associated with aseptic loosening of the cup due to the shortening of the length. The loosening of the cup resulted in chronic inflammation, the development of lesions, or granulation in the surrounding tissue. The osteolysis presented with soft tissue mass or lesions is sometimes neglected as a chronic inflammation-associated pseudotumor. These lesions initially did not display any malignancy but later accumulated and transformed into a tumor mass with vascular lesions and nodules identified as AS in the clinicopathological analysis. Revision surgeries without definitive clinical analysis of the observed chronic lesions could probably be a prognostic factor in the malignant transformations of the latently proliferating tissue lesion into a tumor mass. Only in the case of a 75-year-old man reported by Terrando et al^[61]^ and another case by Zhu et al^[59]^ had presented with a mass in the thigh causing pain and reduced locomotive movement at the time of admission. In both of these cases, there was no prior history of revision of the THA.

It is noteworthy that there had been delays in the early diagnosis of AS in most cases as the malignancy was not observed and thus was misinterpreted as reactive changes or chronic inflammation associated with pseudotumor or granuloma. Secondly, the aseptic loosening and osteolysis requiring revision surgery was the main focus of orthopedics at that time, and tissue biopsy for the granulation or tissue lesion remained scarcely attended. In most circumstances, it is diagnosed at a later stage when the condition of the patient has been severely aggravated with distant metastasis, and the chances for overall survival are marginal. The number of reported cases of AS associated with THA is very few. Thus, the suspicion of AS in THA cases by orthopedics remains largely unconsidered, and the definitive features are also underexplored. Altogether, an alarming suspicion for AS should be considered if a patient displays pain associated with severe osteolysis around the implant, uncontrolled bleeding/hemorrhage, progressively deteriorating status, loss of weight, soft tissue mass, and granulomatous or chronic tissue lesion. In this section, we have collated and evaluated these cases to present the common features of AS associated with THA and raise timely suspicion of AS for treatment. PET-CT scan can aid in the timely evaluation of malignancies and metastatic progression for strategizing treatment regimes. Further, a detailed investigation is warranted to explore the pathogenic association between chronic particle-induced inflammation or soft tissue lesions and the progression of AS.

## 5. Biomedical implant tumorigenesis: vascular prosthesis and autologous graft linked to angiosarcoma

Synthetic arterial reconstruction with Dacron prosthetic material is becoming increasingly common to treat blockage in the arteries and repair of aneurysm. In 1894, Abbe first developed a prosthesis-based arterial reconstruction procedure using a thin glass tube to reunify the femoral artery of a dog.^[62]^ Endovascular aneurysm repair is a subtype of arterial reconstruction method popularly used to treat abdominal aortic aneurysms. With the increasing use of Dacron, polytetrafluoroethylene, and other vascular prostheses to treat aortic aneurysms, a concomitant rise in complications such as endoleaks has also been observed. Notably, treating endoleaks linked to vascular prostheses using percutaneous procedures increases the risk of infection with a reported incidence rate of 0.2% to 0.7%. The gold standard to treat infected vascular prostheses is complete removal of the vascular endograft followed by regrafting.^[63]^ Various experimental investigations were conducted to analyze the carcinogenic characteristics of polymers through subcutaneous implantation in animals that have demonstrated induction of sarcomas.^[9,52,64]^ However, no such direct effect with these materials has been identified in humans. Primary angiosarcoma of the aorta is a rare type of aortic sarcoma, with a handful of cases reported associated with vascular endograft prostheses. An increasing number of reported cases of AS in association with aortic aneurysm surgery using endograft prostheses raises a suspicious relationship between them. The diagnosis of an underlying aortic malignancy can be challenging and frequently delayed as the presenting symptoms can be nonspecific and might be erroneously attributed to more common aortic disease processes. A vast literature search identified 27 cases of synthetic vascular prostheses associated with AS development, summarized in Table 3.

**Table 3:** Review of literature for cases of vascular prosthesis associated with AS development.

Author	Year	Age/sex	Vascular prosthesis	Interval to AS develop-ment (yr)	Tumor site	Tumor status	Treatment/management	Outcome
Fehrenbacher et al^[65]^	1980	67/M	Dacron graft	12	Infrarenal aorta	Metastasized to liver and lungs	Surgery, chemotherapy	Died 7 mo after surgery
Weiss et al^[66]^	1991	56/M	Dacron graft	3	Infrarenal aorta	Metastasized to liver	Surgery, chemotherapy	Alive with disease 11 mo after surgery
Dargent et al^[67]^	1997	61/M	Dacron graft	ND	Iliac artery	Metastasized to brain, lungs, liver, spleen, lymph nodes, and bone marrow	Surgery	Died 9 d after admission
Ben-Izhak et al^[68]^	1999	71/M	Dacron graft	8	Iliac artery	No metastasis reported	Surgery	Died 6 mo after diagnosis
Okada et al^[69]^	2004	50/M	Dacron graft	17	Ascending aorta	Metastasized to brain	No	Died 2 wk after admission
Umscheid et al^[70]^	2007	50/M	Stent and Dacron graft	5	Infrarenal aorta	Metastasized to lung	Surgery, chemotherapy	Died 24 mo after surgery
Almeida et al^[71]^	2011	60/M	Dacron graft	9	Right atria	Metastasized to lung, left renal, and adrenal	Chemotherapy, radiotherapy	Died after 3 wk after diagnosis
Schmehl et al^[72]^	2011	77/ND	Dacron graft	7	Infrarenal aorta	No metastasis reported	Surgery, chemotherapy	ND
Brendle et al^[73]^	2011	84/M	Vascular graft	8	Infrarenal aorta	Metastasized to lymph node, bone	Surgery	Died few weeks after surgery
Fatima et al^[74]^	2012	57/M	Dacron graft	6	Infrarenal aorta	Metastasized to liver, lung, and lymph nodes	Surgery	Died 2 mo after surgery
Stewart et al^[75]^	2013	86/M	Ancure aortic graft	9	Iliac artery	Bone and skin metastasis	Surgery	Died 3 mo after surgery
Fenton et al^[76]^	2014	72/M	AneuRx stent graft	6	Iliac artery	Bone metastasis	Palliative corpectomy, chemotherapy	Died 1 mo after diagnosis
Kimura et al^[77]^	2015	78/M	Dacron graft	16	Distal aortic arch	No metastasis reported	Surgery	Died 2 mo after treatment
Bader et al^[78]^	2016	72/M	Vascular graft	7	Proximal tibia	No metastasis reported	Amputation of leg	ND
Milite et al^[79]^	2016	60/M	PTFE graft	7	Infrarenal aorta	Peritoneal metastasis	Surgery	Died 1 mo after surgery
Kamran et al^[80]^	2016	72/F	Stent graft	0.25	Infrarenal aorta	Metastasized to left renal vein and gluteal muscle	Chemotherapy	ND
Kamran et al^[80]^	2016	69/M	Vascular graft	8	Infrarenal aorta	No metastasis reported	Chemotherapy, radiotherapy	Alive 9 mo after treatment
Tiwari et al^[81]^	2016	64/M	Dacron graft	7	Abdominal aorta	No metastasis reported	Chemotherapy	ND
Cherchi et al^[82]^	2017	64/F	Vascular graft	4	Left popliteal fossa	No metastasis reported	Surgery	Died 17 d after diagnosis
Chetouani et al^[83]^	2017	81/M	Vascular graft	1	Femoral artery	ND	ND	ND
Dietl et al^[84]^	2018	59/M	Stent graft	15	Neck	Metastasized to neck, chest, abdomen, pelvis and lungs	Surgery	Died due to respiratory failure after admission
Yu et al^[85]^	2019	68/M	PTFE graft	4	Infrarenal aorta	Metastasized to liver and vertebra	Surgery, chemotherapy	Alive with disease 2 mo after surgery
Presacco et al^[12]^	2020	84/M	PTFE graft	5	L3 vertebra	No metastasis reported	Surgery	Died within 2 mo of admission
Derouane et al^[86]^	2020	68/M	Dacron graft	3.5	Iliac artery	No metastasis reported	Surgery, chemotherapy	Died 5 wk after diagnosis
Almajan et al^[87]^	2021	69/F	Endurant stent graft	3	Abdominal aorta	Metastasized to left iliac fossa lymph nodes, soft tissues of the right buttock, distal left thigh, pelvic bones, and left femur	Surgery	Died 3 mo after diagnosis
Takamura et al^[88]^	2021	82/F	Vascular graft	4	Thoracic aorta	No metastasis reported	Antibiotics	Died 2 mo after diagnosis
Arts et al^[89]^	2022	79/F	Vascular graft	9	Right popliteal fossa	Metastasized to lung, bone, and lymph nodes	Surgery, adjuvant chemotherapy	Died 8 mo after diagnosis

AS = angiosarcoma, F = female, M = male, ND = not disclosed, PTFE = polytetrafluoroethylene.

Development of AS linked to vascular endoprosthesis has been reported in various arterial aneurysms possibly due to the polyester material of the endograft. Out of the 27 cases of aneurysm repair, a total of 13 cases of abdominal aorta, 4 cases of femoral artery, 6 cases of femoropopliteal, and 3 cases of popliteal artery reconstruction had progressed into angiosarcoma. Takamura et al^[88]^ reported the only case of identification of AS proximal to the thoracic endovascular aneurysm repair performed 4 years ago. Interestingly, most AS cases observed are reported in males, with a few cases in females (N = 5). Also, all the patients were of age more than 50 years. Based on the observation, a multimodal evidence-based investigation is required to validate whether a significant correlation exists between the demographic characteristics (age and gender) and AS development proximal to vascular endograft.

The latency period highly varies (3 months to 17 years) from case to case relating to their clinical history. The lowest latency period reported by Kamran et al^[80]^ and Chetouani et al,^[83]^ past medical conditions have been a major contributing factor leading to a shorter latency period. Thus, the duration of AS development due to failure of vascular endograft or associated complications such as endoleaks and thrombosis could be influenced by multiple factors, with past medical conditions being one of the significant contributors. The plausible pathogenic progression of AS linked to vascular prostheses may involve the formation of a fibrous capsule induced by chronic inflammation around the endograft, followed by increased fibrosis and vascular proliferation.

Early identification of AS associated with synthetic endoprosthesis remains challenging as the presenting symptoms would mimic those of thromboembolic disease and atherosclerosis and are most often misinterpreted. The presence of intraluminal mass might warrant a high level of clinical suspicion for AS. However, in many cases, this mass is also neglected and is considered a pseudotumor or inflammation-associated granuloma.

On contrary to the tumorigenic potential of synthetic vascular endograft made up of Dacron and stent in triggering the pathogenic development of AS in many clinical cases. Here we also report 8 cases (Table 4) of AS development found to be closely associated with autologous saphenous endograft implanted during a past bypass surgery to treat aneurysm. Pathogenic mechanisms involved in these cases may probably differ from those of the synthetic Dacron graft associated AS. The oncogenic effect contributed by the synthetic vascular graft due to foreign body reaction could not be attributed in cases of autologous vein graft.

**Table 4:** Review of literature for cases of autologous vascular endoprosthesis associated with AS development.

Author	Year	Age/sex	Vascular prosthesis	Interval to AS development (yr)	Tumor site	Tumor status	Treatment/management	Outcome
Kogon et al^[90]^	1998	70/M	Saphenous vein graft	13	Right popliteal artery	No metastasis reported	Surgery, radiotherapy	ND
Nocturne et al^[91]^	2010	81/M	Saphenous vein graft	15	Popliteal artery	No metastasis reported	Radiotherapy	Died 7 mo after diagnosis
Morris et al^[92]^	2018	69/M	Saphenous vein graft	2.5	Left femur	Metastasized to lung and bone	Surgery, radiotherapy	Died 3 mo after surgery
Villaescusa Catalan et al^[93]^	2019	74/M	Saphenous vein graft	8	Femoral artery	No metastasis reported	Surgery	Died 2 mo after surgery
Hartvigson et al^[94]^	2019	72/M	Saphenous graft	18	Right atrium	Metastasized to lung	Surgery, adjuvant, and palliative radiotherapy	Multiple local recurrences and died 21 mo after surgery
Werra et al^[95]^	2021	83/M	Saphenous vein graft	4	Right popliteal fossa	Oral and right shoulder metastases	Surgery	Died 1 yr after initial admission
Veterano et al^[96]^	2022	58/M	Saphenous vein graft	9	Femoral artery	Metastasized to the brain, lung, and liver	Surgery	Died 2 mo after diagnosis
May Lee et al^[13]^	2022	84/M	Saphenous vein graft	3	Medial aspect of the knee	No metastasis reported	Palliative chemo-radiotherapy	Alive with disease 20 mo after initial diagnosis

AS = angiosarcoma, M = male, ND = not disclosed.

In absence of any foreign body involvement the development and prognosis of AS vicinal to saphenous vein graft could results as a consequence of local chronic inflammation, surgically altered environment, exposure to surgical equipment (subcutaneous stitching material or metallic clips), or due to altered blood flow. Some authors have hypothesized the fibrotic process due to local tissue reaction as a potential prognostic mechanisms in the development of AS. Out of the 8 cases of autologous graft associated AS, 5 cases are of femoropopliteal artery and 2 cases of popliteal artery aneurysm. While several cases of autologous graft associated AS was observed in femoropopliteal and popliteal bypass surgery. Hartvigson et al^[94]^ in 2019 reported the first unique case of AS originating from saphenous vein graft used for treatment of coronary artery aneurysm. The latency period in this case was 18 years after the graft was inserted.

Furthermore, several cases of AS have been found to be associated with arteriovenous fistulae in renal dialysis patients without kidney transplant.^[97–100]^ Altogether with several emerging clinical cases of AS closely linked to arteriovenous or autologous grafts which are completely biological and no involvement of metallic foreign body or biomedical implants-mediated tumorigenesis. These cases indicates that the pathogenic progression of AS in patients with synthetic biomedical implants or metallic foreign body could be multifactorial. Thus presence of foreign body in these cases could be a triggering factor initiating tissue damage, local chronic inflammation followed by extensive fibrosis. Either it be the case of biologic grafts or synthetic/metallic orthopedic and vascular grafts, local chronic inflammation could be hypothesized as the trigger initiation point that slowly culminates into AS. Therefore, a thorough multimodal approach is required to unveil the exact mechanism involved and establish the substantial contribution of various factors to the pathogenesis of AS. A detailed understanding of etiology, diagnostic features and mechanistic progression could aid in early identification before it has metastasized and a multifocal therapeutic treatment approach could be initiated.

## 6. Conclusion

Angiosarcoma is a rare, highly aggressive soft tissue sarcoma associated with a high rate of local recurrence and metastasis. Poor prognosis limits the establishment of a direct etiological association between metallic foreign body material or biomedical implants and the development of AS and may prove circumstantial. In the past couple of centuries, the accrual of reported cases of AS in patients proximal to a retained metallic foreign body or biomedical implants without any history of other risk factors has raised a clinical suspicion of a plausible pathogenic link. Our study, for the first time, reviews all the cases, though rare, outlining the plausible pathogenic potentials of metallic foreign bodies and vascular and orthopedic prostheses in evoking the development of angiosarcoma. With increasing etiological evidence of AS development at the site of the preimplanted biomedical prosthesis, we aim to create an awareness of the possibility of masked AS being suspected for unusual findings. Revision surgery due to an aneurysm should be monitored with high clinical suspicion through PET-CT or PET-MRI and angiography for atypical presentation or masses for early diagnosis of suspected AS. A multimodality approach involving chemotherapy, radiotherapy, surgery, and advanced immunotherapeutics may aid in effectively managing and evading the metastatic progression of the disease with improved survival.

## Author contributions

**Conceptualization:** Ramy Samargandi.

**Data curation:** Ramy Samargandi.

**Formal analysis:** Ramy Samargandi.

**Investigation:** Ramy Samargandi.

**Writing – original draft:** Ramy Samargandi.

**Writing – review & editing:** Ramy Samargandi.
